# Supplementary material for: Examining an Altruism-Eliciting Video Intervention to Increase COVID-19 Vaccine Intentions in Younger Adults: A Qualitative Assessment Using the Realistic Evaluation Framework
Source: Vaccines (Basel). 2023 Mar 11;11(3):628. doi: 10.3390/vaccines11030628 (PMC10056235; doi:10.3390/vaccines11030628)
Supplement: Supplementary file 1 [file vaccines-11-00628-s001.zip › S1 Interview Guide.pdf]

Interview Guide

Introduction

1. Explain that we are going to show them a video in both English and French related to COVID-19 vaccination that was developed for a study. Afterwards, we will ask them their thoughts about it.
2. Ask if participants have any questions.
3. Explain context of when video was done

[SHOW VIDEO IN ENGLISH AND FRENCH]

The following are major themes to be addressed in the focus group. Participants will be able to ask the moderator for clarification about any of the questions or any terms used.

Context

1. **Context** – How do you think the current situation with COVID-19 impacts vaccination and now uptake of additional/booster doses?
2. **Context** – How do you think the situation is different from 2021, when vaccines for COVID-19 were first introduced?

Mechanism

3. **Mechanism** – How do you think the video might impact someone's decisions about vaccination (back in 2021 vs. now [C])?
    a. [If needed explain that we designed the video to have an altruistic message, i.e., the desire to help others without expecting personal benefit]
4. **Mechanism** – How would you improve the message presented in the video?
5. **Mechanism -** If you were making a video to get people your age to vaccinate, what would be the message you would try to send?

Outcome

6. **Outcome** – In our study, we found that people who had decided against receiving the vaccine were not likely to change their decision after watching the video. What do you think could be blocking them from considering vaccination?
7. **Outcome** – In a study, we found that people who were undecided or had not thought about vaccination were more likely to want to vaccinate after viewing the video. What do you think might have made them change their decision?

Conclusion

1. Thank you for participating in this discussion and for your helpful feedback. Are there any other comments you have about what we discussed, or questions about our study?
