# Supplementary material for: Examining an Altruism-Eliciting Video Intervention to Increase COVID-19 Vaccine Intentions in Younger Adults: A Qualitative Assessment Using the Realistic Evaluation Framework
Source: Vaccines (Basel). 2023 Mar 11;11(3):628. doi: 10.3390/vaccines11030628 (PMC10056235; doi:10.3390/vaccines11030628)
Supplement: Supplementary file 1 [file vaccines-11-00628-s001.zip › S2 Themes and Quotes.pdf]

Themes and quotes

Context	Sample Quotes
Attitudes and beliefs about health policies	"I hated that the government was imposing these restrictions, I found it to be very divisive. Probably helpful in getting people vaccinated, but not right on a human rights perspective, from my view. That's a different story, but I guess, for me, that didn't have an impact, I could have lived without going to restaurants, and not taking the metro, and all that" (Fully vaccinated, P3)
Perceptions about media and public health messaging	"I would say that I think that specific video you showed would have a less of an effect today compared to a year ago, and not specifically because of the content of the theme of altruism that was used, but rather it sounds a lot like many of the other messages we've heard from the government, for example, about getting vaccinated, and then people have probably heard that message over and over and over again. So at this point when they kind of feel like a similar message to them, it doesn't really affect them as much as it would probably a year ago." (Fully vaccinated, P4)
Return to normalcy	"I guess I've also noticed that the conversation doesn't really revolve around like which dose you're at and are you going to get the next ones, it's more about the measures and getting them lifted, yeah." (Boosted, P4)
Mistrust in government and institutions	"But in terms of how the government has handled things, even all the commercials that I saw on TV, all the information that was provided, there was no transparency. And to me, that reflects society. For decades, that's been going on this way forever. There is lacking transparency. And without transparency, there is no trust. So then things get [inaudible]. One day here, this is one thing in the news. Then they will get to something else. There was no continuity, so there is no trust." (Unvaccinated, P2)
**Mechanism**	
Perceived susceptibility	"Fundamentally, at least for me, the reason was basically it shouldn't-- I'm the youngest person here. I'm in my 20s. So I don't think that it's even necessary for me to-- I don't think the

	coronavirus is a threat at all, right, if you look at it in the aggregate sense. And I did get COVID, I think, twice. But it's not a threat for me." (Unvaccinated, P3)
Protecting vulnerable persons	"I believe that society or anything involved with society is a collective effort. We are all responsible for each other" (Unvaccinated, P2)
Perceived vaccine efficacy	"I would say it also has a less effect today because I'm no medical expert but it seems like the vaccine acts more as a therapeutic to prevent serious illness, so people who want to prevent getting-- from serious illness or death, right? But it doesn't really necessarily protect you from getting sick like a typical vaccine would, like a chickenpox vaccine, which prevents you from getting sick to begin with." (Fully vaccinated, P3)
Perceived harms	"My point is this. The vaccine came out way too quickly. There was not enough information. I would go look up on Pfizer. I went and looked up on Moderna to see what the side effects were. And there are side effects. And even though the percentage could be very small, you don't know if you're going to be the person that gets the side effect. It's a fact. It's a reality when you get anything done, any medical treatment." (Unvaccinated, P2)
Social influence	"it's a couple that decided to not get vaccination, is they quoted religion. I don't exactly know-- I think they're Mormon, I'm not exactly sure, please don't quote me on that. But it's something for religious reasons, or at least that's what they told me as a reason, and they are pretty devote in their religion." (Fully vaccinated, P2)
Anti-vaccination beliefs	"I feel like regardless of what video they saw or what information they got, when someone has a set opinion about something, they're unlikely to change it, despite if they're getting statistics or personal anecdotes or information. And so this could apply to anything, not just vaccination, in terms of just like personal believes and opinions. And so no matter what you do it's going to be really difficult to change it, I think." (Fully vaccinated, P4).
Perceived severity	"A lot of people I feel as though were not afraid to get COVID, just because they can get the flu and have way worse symptoms than just was-- was watching Netflix in bed for today." (Boosted, P6)

Individualism	“I would certainly say that, that obviously, I've heard of a certain population that have, let's say, a lower immune system that are still very cautious, but I think they are cognizant that the world certainly seems to have moved in a different direct, that they need to take precautions into their own hands.” (Fully vaccinated, P2)
**Intervention-specific suggestions**	
Content	“I would say that I think that specific video you showed would have a less of an effect today compared to a year ago, and not specifically because of the content of the theme of altruism that was used, but rather it sounds a lot like many of the other messages we've heard from the government, for example, about getting vaccinated, and then people have probably heard that message over and over and over again. So at this point when they kind of feel like a similar message to them, it doesn't really affect them as much as it would probably a year ago.” (Fully vaccinated, P4)<br><br>“I think maybe like the combination of the graphic imagery with, maybe, some sort of simplified yet believable statistic to kind of go hand in hand, because maybe sometimes, if you're focusing too much on the anecdote, like the video had three particular examples, it makes it more personal, but then, at the same time, it might remove from the vastness of the effects. So having an extra statistic in the video that showed like, "And, by the way, these three examples, they happened this amount," to kind of give you a different aspect to the impact that it had.” (Boosted, P4)
Design	“And it's like, I think, what would be really interesting in the video is, maybe, have nurses and all these people who have been central in maintaining the health and safety of so many people to hear their perspective of the situation.” (Boosted, P6)<br><br>“Or I would have called that video almost like emotional blackmail or something. You're not talking about science. You're not talking about numbers. It's just all about feelings and about community, which is nice, but that's the problem. I don't want to hear about that when we're

	talking about science. I want to hear about science because I'm not a scientist.” (Unvaccinated, P1)
